# Consistency mapping of 16 lymph node stations in gastric cancer by CT-based vessel-guided delineation of 255 patients

**DOI:** 10.18632/oncotarget.18407

**Published:** 2017-06-08

**Authors:** Shuhang Xu, Lingling Feng, Yongming Chen, Ying Sun, Yao Lu, Shaomin Huang, Yang Fu, Rongqin Zheng, Yujing Zhang, Rong Zhang

**Affiliations:** ^1^ Department of Ultrasound, The Third Affiliated Hospital of Sun Yat-Sen University, Guangzhou 510630, China; ^2^ State Key Laboratory of Oncology in Southern China, Guangzhou 510060, China; ^3^ Department of Radiation Oncology, Sun Yat-Sen University Cancer Center, Guangzhou 510060, China; ^4^ Department of Gastric Surgery, Sun Yat-Sen University Cancer Center, Guangzhou 510060, China; ^5^ Guangdong Province Key Laboratory of Computational Science, School of Data and Computer Science, Sun Yat-Sen University, Guangzhou 510006, China; ^6^ Department of Statistical Science, Sun Yat-Sen University School of Mathematics, Guangzhou 510275, China; ^7^ Department of Radiology, Sun Yat-Sen University Cancer Center, Guangzhou 510060, China

**Keywords:** gastric cancer, lymph node stations, mapping, computer software, clinical target volume

## Abstract

In order to refine the location and metastasis-risk density of 16 lymph node stations of gastric cancer for neoadjuvant radiotherapy, we retrospectively reviewed the initial images and pathological reports of 255 gastric cancer patients with lymphatic metastasis. Metastatic lymph nodes identified in the initial computed tomography images were investigated by two radiologists with gastrointestinal specialty. A circle with a diameter of 5 mm was used to identify the central position of each metastatic lymph node, defined as the LNc (the central position of the lymph node). The LNc was drawn at the equivalent location on the reference images of a standard patient based on the relative distances to the same reference vessels and the gastric wall using a Monaco® version 5.0 workstation. The image manipulation software Medi-capture was programmed for image analysis to produce a contour and density atlas of 16 lymph node stations. Based on a total of 2846 LNcs contoured (31–599 per lymph node station), we created a density distribution map of 16 lymph node drainage stations of the stomach on computed tomography images, showing the detailed radiographic delineation of each lymph node station as well as high-risk areas for lymph node metastasis. Our mapping can serve as a template for the delineation of gastric lymph node stations when defining clinical target volume in pre-operative radiotherapy for gastric cancer.

## INTRODUCTION

Gastric cancer (GC) is the fourth most common malignancy in the world, and half of all cases occur in East Asia [1]. In China, GC is the second most common malignancy [2]. Although surgical resection is the central curative treatment, neoadjuvant radiotherapy has played an increasingly prominent role in GC, particularly for locally advanced disease [3, 4]. Several studies have demonstrated that pre-operative chemoradiotherapy for resectable GC is associated with a significant survival benefit compared with surgery alone [5–9].

Lymph node (LN) metastasis is the main metastatic pathway of GC, and the LN metastasis rate can be up to 10–20% in early GC [10]. The first English edition of the general rules of the Japanese Research Society for Gastric Cancer (JRSGC) classifies regional LNs into 16 stations by location, which has been widely accepted and adopted in many countries [11]. In 2009, Matzinger et al. established treatment guidelines for neoadjuvant radiation of GC and suggested clinical target volumes for elective lymph node stations (CTV_electives_) [12]. However, difficulty and confusion among radiation therapists in pre-operatively determining CTV_electives_ has also been reported [13, 14]. As radiation treatment fields become increasingly conformal to limit doses to normal critical structures, there is an urgent need to accurately identify evidence-based CTV definitions for neoadjuvant radiotherapy of GC. However, no consensus has been reached for this topic. Thus, variability in CTV delineation can be large without a universally accepted standard for mapping LN stations.

In 2009 and 2013, Matzinger et al. and Jansen et al. developed contouring atlases for the gastric LN stations [12, 13]. However, the atlases were created according to expert opinions instead of using actual data regarding the distribution of metastasis to LNs in GC patients. Moreover, these studies did not provide the metastasis-risk density for each LN station.

At present, medical image processing software is widely used [15, 16], especially in the fields of radiology and radiotherapy. In the current study, we retrospectively reviewed the initial images and pathological reports of 255 GC patients with lymphatic metastasis, utilizing computer technology to identify the location and metastasis-risk density of 16 LN stations by computed tomography (CT)-based vessel-guided delineation and to refine the CTV delineation for neoadjuvant radiotherapy of GC.

## RESULTS

**Figure 1:**
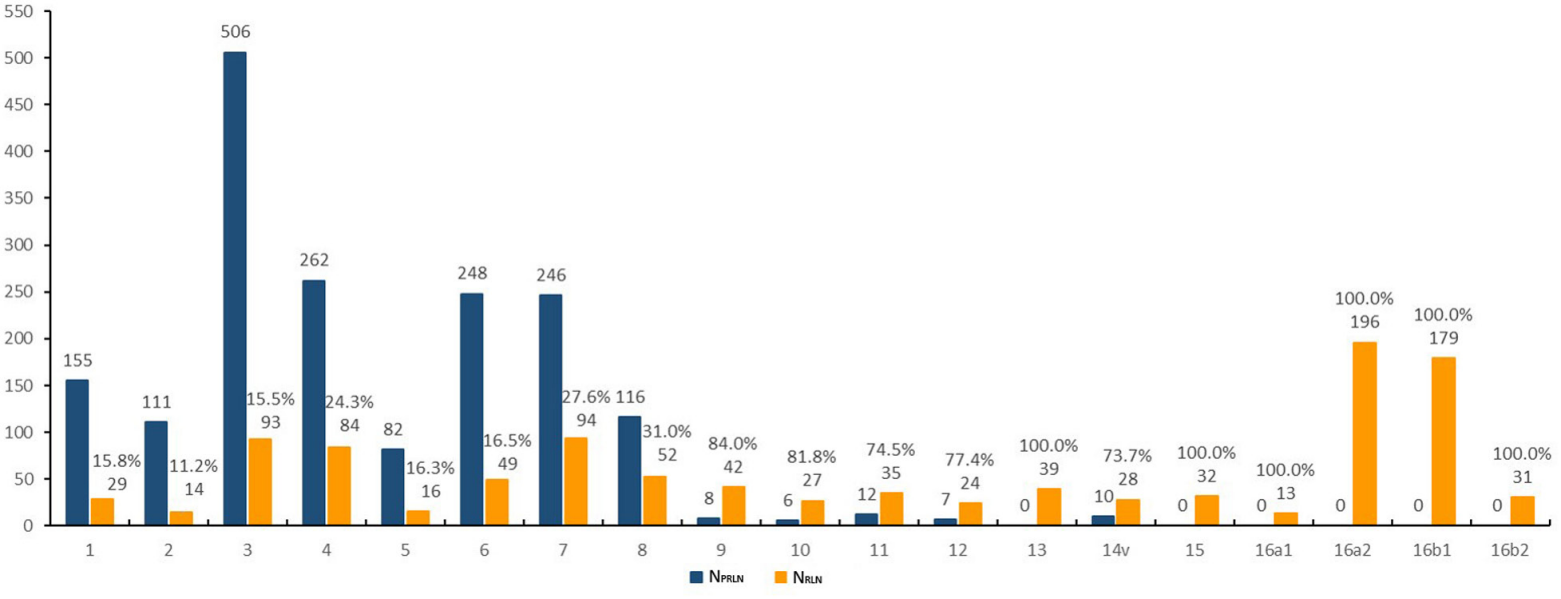
Distribution of 2846 metastatic lymph nodes N_PRLN_: the number of pathological- and radiological-positive lymph nodes; N_RLN_: the number of radiological-positive lymph nodes.

The distribution of the 2846 examined LNcs is shown in Figure 1. 1769 (62.2%) were described as pathological- and radiological-positive lymph nodes (PRLNs) and 1077 (37.8%) as radiological-positive lymph nodes (RLNs).

**Figure 2:**
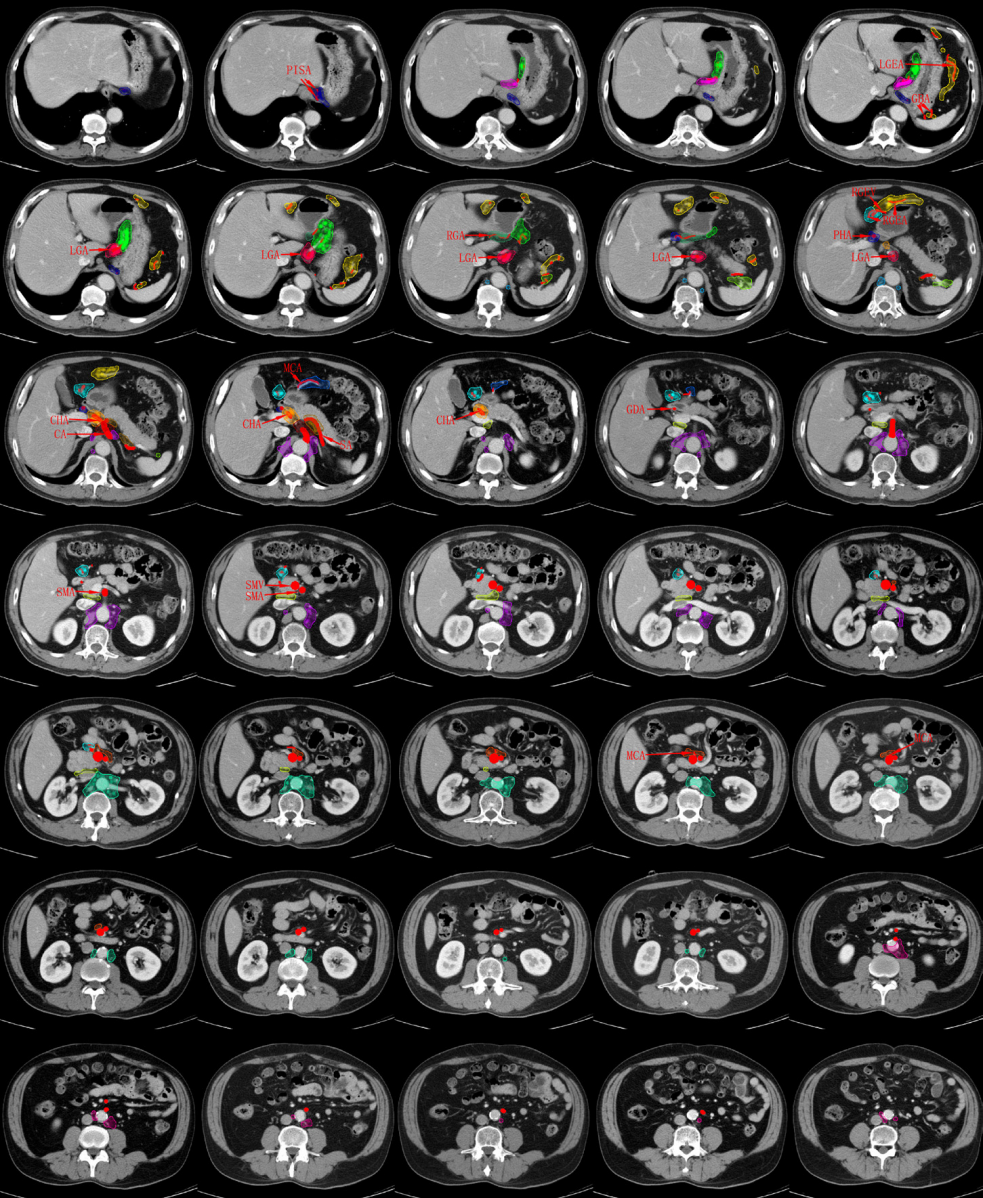
Consistency mapping of 16 lymph node stations in gastric cancer by CT-based vessel-guided delineation of 255 patients In total, 35 representative axial CT images were selected moving in the cranial to caudal direction in 5 mm slices. PISA: phrenica inferior sinistra artery; LGA: left gastric artery; RGA: right gastric artery; GBA: gastricae breves artery; LGEA: left gastroepiploic artery; RGEA: right gastroepiploic artery; RGEV: right gastroepiploic vein; PHA: proper hepatic artery; CHA: common hepatic artery; CA: celiac artery; SA: splenic artery; MCA: middle colic artery; GDA: gastroduodenal artery; SMA: superior mesenteric artery; SMV: superior mesenteric vein.

Figure 2 depicts the radiographic delineation and metastasis risk density of the 16 LN stations on a standard patient.

The right pericardial LNs run along the ascending branch of the left gastric artery, located in the narrow anatomic space between the gastric cardia and liver edge, extending inferiorly by the lesser curvature LNs and in the upper part of the nodes along the left gastric artery. The high-risk metastasis region is located between the gastric cardia and the ascending branch of the left gastric artery.

The left pericardial LNs run along the corresponding esophageal branch of the left inferior phrenic artery. The volume is medial to the gastric fundus and superior to the hemidiaphragm.

The lesser curvature LNs run along the branches of the left gastric artery and along the 2^nd^ branch and distal part of the right gastric artery, which are defined laterally by the gastric body, superiorly by the right pericardial LNs and inferiorly by the suprapyloric LNs. The metastatic risk density is higher around the branches of the left gastric artery than around the right gastric artery.

The greater curvature LNs run along the short gastric vessels, the left gastroepiploic vessels, and the 2^nd^ branch and distal part of the right gastroepiploic artery. This LN basin is defined laterally by the gastric body and posteriorly by the spleen and splenic hilum LNs.

The suprapyloric LNs, which lie directly superior to the gastric pylorus, run along the 1^st^ branch and the proximal part of the right gastric artery. The suprapyloric LNs are bordered on the right side by the inferior portion of the lesser curvature LNs.

The infrapyloric LNs run along the first branch and proximal part of the right gastroepiploic artery down to the confluence of the right gastroepiploic vein and the anterior superior pancreatoduodenal vein. The infrapyloric LNs lie inferior to the gastric pylorus and anterior to the pancreatic head, and the lowest level is located in the right front corner of the superior mesenteric LNs.

The left gastric LNs run along the trunk of the left gastric artery between its root and the origin of its ascending branch, the area located superior to the celiac axis and inferior to the right pericardial LNs, merging with the lesser curvature LNs. The metastatic risk appears higher on the left side and anterior to the left gastric artery than on the right side of the vessel.

The common hepatic LN station consists of a defined volume around the vessel and is bordered posteromedially by the celiac LNs and laterally by the hepatoduodenal LNs.

The area containing the celiac LNs surrounds the celiac artery, starting from its origin from the aorta to its termination, where it branches off into the common hepatic artery, the left gastric artery, and the splenic artery. Our study found that the LN basins are concentrated in the anterior half of the celiac artery.

The splenic hilar LN basin covers all the splenic hilum vasculature, lies posterior to the greater curvature LNs and posterolaterally to the spleen, and represents the area between the spleen and pancreatic tail.

The splenic artery LN basin starts from the origin of the splenic artery to the end of the pancreatic tail. The high-risk metastatic region is located in the proximal part of the splenic artery.

The hepatoduodenal ligament LNs lie along the proper hepatic artery, the common bile duct, and the portal vein and are located between the confluence of the right and left hepatic ducts and the upper border of the pancreas, in the anterior surface of the main portal vein.

The posterior pancreatic LNs lie on the posterior surface of the pancreatic head and anterior to the paraaortic LNs, extending to the portacaval space. It appears that the high-risk metastatic region is located in the higher position of this volume.

The superior mesenteric vein LNs and middle colic vessel LNs run along the surface of the superior mesenteric vessels and the middle colic vessels, respectively, inferior to the gastric wall.

The paraaortic LNs are divided into the 16a1, 16a2, 16b1 and 16b2 stations. The 16a1 station is located in the diaphragmatic aortic hiatus, on the left and right side of the aorta. The 16a2, 16b1, and 16b2 paraaortic LNs extend superiorly from the upper margin of the origin of the celiac artery to the bifurcation of the iliac artery, within the surrounding region and immediately adjacent to the aorta. Our study found that the 16a2 and 16b1 stations were most involved in LN metastasis, followed by the 16b2 station, while the 16a1 station was seldom involved.

## DISCUSSION

In the present study, we retrospectively reviewed the initial images and pathological reports of 255 GC patients with lymphatic metastasis. After contouring 2846 LNcs (31–599 per LN station) on CT images, we created a density-distribution map of 16 LN drainage stations of the stomach.Importantly, these stations can reflect the true state of LN metastasis in patients. This is the first study to take advantage of data-processing software to analyze the distribution and high-risk areas of LN metastasis.

CT scans of the abdomen are mandatory for precise preoperative tumor and node metastasis staging [17, 18]. Previous studies have reported that the diagnostic accuracy of LN metastasis in gastric cancer has varied from 54–84% [19], while the sensitivity has varied from 48–91% [20, 21]. We used a 64-slice spiral CT with a scanning layer thickness of 1-mm pitch to ensure that all LNs were detected. The number of LNs in each station ranged from 31–599 in our study, which is sufficient to create a LN distribution in the software.

Previous studies that have developed contouring atlases of the gastric LN stations based on expert advice have several key differences compared with our study. For example, the atlas created by Oscar M described the areas corresponding to the 4^th^ and 5^th^ LN stations as covering most of the gastric area [12], which may lead to radiation-related side effects. Additionally, the atlas created by Jennifer Y suggested that the areas corresponding to the 2^nd^ and 4^th^ LN stations cover all the fat space between the gastric wall and abdominal wall [22], which is a large area around the vessels. Our present study indicates that a definition area should also describe the distance between vessels and corresponding organs and the size of the surrounding adipose space. For example, for the 1^st^–6^th^ perigastric LN stations, we suggest that the volume should cover the fat space between the corresponding vessels and the gastric wall, whereas the volume can vary according to the surrounding adipose space for the other side of the vessels.

A previous study also suggested that CTV_elective_ should be defined by a 5-mm margin around the corresponding vessels [12]. However, our study indicates that differences exist among the different LN stations with respect to the distance between vessels and corresponding organs and the size of the surrounding fat space. Moreover, metastatic risk is also an important indicator of the margin range of corresponding vessels. For example, because the lesser curvature station has the highest metastatic rate of the stations, we suggest the volume of the 3^rd^ LN station should be defined by a 10–20 mm margin around the corresponding vessels or should cover the overall adipose gap of the lesser curvature if the area is too small. The 7^th^ LN station also has a high risk of metastasis, and the volume should cover a 5–15 mm margin or the overall adipose gap along the left gastric artery. For the 1^st^, 2^nd^, 4^th^, 5^th^, 6^th^, 8^th^–12^th^, 14^th^ and 15^th^ LN stations, we suggest the volume should be defined by a 5–10 mm margin around the corresponding vessels. For the 16^th^ LN station, because 16a2 and 16b1 are more frequently involved and with a large adipose gap around the aortas, we suggest that the volume should be defined by a 20 mm margin around the aorta, and a 10 mm margin around the aorta should be used for the 16a1 and 16b2 stations.

Some suggestions for the delineation of CTV_elective_ can be made based on our mapping. For the 13^th^ LN station, we suggest that the portacaval space should also be included because LN metastasis is involved in this space. In addition, it is notable that the range of the 6^th^ LN station down to the confluence of the right gastroepiploic vein and the anterior superior pancreatoduodenal vein is bordered by the 14^th^ LN station. Although we suggest the delineation of CTV_elective_ based on vascular structure, some vessels may not be clearly detected by CT when a patient is too thin or as a result of tumor invasiveness. Since radiation treatment fields become increasingly conformal nowadays, accurate radiotherapy can increase the treatment efficiency and also limit doses to normal critical structures. Our mapping can thus be a reliable reference for deciding on the volume of prophylactic irradiation for LN stations.

Our study has limitations. First, although we aimed to identify a standard patient whose abdomen CT reflected most cases, there are anatomical differences and abdominal blood vessel variations between standard patients and GC patients. Second, 37.8% of the LNs were radiologically diagnosed without a pathological result, which could have produced false positives and false negatives in our study.

To the best of our knowledge, this is the first study to date that uses mathematical software technology to identify the true state of LN metastasis in GC patients by CT-based vessel-guided delineation of metastatic LNs. The mapping presents a detailed radiographic delineation of each LN station and identifies high-risk areas for LN metastasis in the 1^st^–16^th^ LN stations, which can serve as a reliable template for the delineation of gastric LN stations when pre-operative radiotherapy for GC is planed. Our mapping can thus help reduce inter-observer variation in CTV delineation of LN stations. Further studies should concentrate on the different high-risk areas of LN metastasis in the three portions of GC, which may provide the basis for individualized GC treatment.

## MATERIALS AND METHODS

### Patients

Between July 2012 and June 2013, the records of 255 patients with newly diagnosed GC were retrospectively reviewed. During this period, 643 GC patients were admitted to the department of gastric and pancreatic surgery in Sun Yat-sen University Cancer Center. Of these 643 patients, 255 patients met the following inclusion criteria: (a) No history of LN tuberculosis, lymphoma or other diseases resulting in enlarged LNs. (b) No history of gastrectomy. (c) Initial CT scan performed in the supine position using intravenous contrast with a 1-mm slice, with the scan area including the diaphragmatic domes to the common iliac artery bifurcation. (d) For patients who underwent gastrectomy and D1+/D2 LN dissection, no neoadjuvant therapy with more than 15 LNs resected and at least 1 PRLN. Postoperative pathologic reports were used to obtain detailed information on the number of positive nodes and to divide them into subgroups. LNs were considered positive for metastasis in radiology when meeting the following criteria: having a short-axis diameter larger than 6 mm for perigastric LNs and larger than 8 mm for extraperigastric LNs, especially nodes of a rounded shape and enhancement on contrast-enhanced CT that were sometimes necrotic [23]. (e) For patients who had not underwent LN dissection, RLNs could be observed in CT images. RLNs have a rounded shape and are necrotic or multinodular confluent on initial CT or show increased/decreased LN size in follow-up CT scanning after chemotherapy [20, 24, 25]. Two radiologists with 15 and 3 years of work experience within the gastrointestinal specialty supervised study enrollment.

LN resection category was dependent on anatomic landmarks. All resected LNs were submitted for histopathologic examination on a nodal group basis. The anatomical definitions of LN stations at surgery were also based on the 3^rd^ classification of the Japanese Gastric Cancer Association (JGCA) [26]. N staging was assessed using the 7^th^ edition UICC classification [27]. LN stations 1–12 and 14v are defined as regional stations. The 13^th^, 15^th^, and 16^th^ LN stations are considered distant stations, and metastasis to these node stations is classified as M1.

Patient, treatment, and tumor characteristics are listed in Table 1.

**Table 1:** The clinicopathologic features of 255 gastric cancer patients

Characteristics		No. patients (%)
Sex	Male	164 (64.3)
	Female	91 (35.7)
Age	Median	57
	Range	26-81
Location of tumor	Upper 1/3	69 (27.1)
	Middle 1/3	79 (31.0)
	Lower 1/3	93 (36.5)
	The whole stomach	14 (5.5)
Tumor size(cm)	<3 cm	22 (8.6)
	≥3, ≤5 cm	132 (51.8)
	>5 cm	101 (39.6)
No. dissected LNs	Median	28
	Range	15-79
No. pathological positive LNs	Median	10
	Range	1-70
Staging*	IB	4 (1.6)
	IIA	9 (3.5)
	IIB	5 (2.0)
	IIIA	30 (11.8)
	IIIB	60 (23.5)
	IIIC	120 (47.1)
	IV	27 (10.6)

*The seventh edition of the AJCC (American Joint Committee on Cancer) staging system.

### MSCT

CT examinations were performed using a 64-slice spiral CT (Aquilion TSX-101A, Toshiba Medical System, Tokyo, Japan). In the entire cohort of 255 patients, the imaging process was performed according to a standard imaging protocol. All patients received 600–800 ml of water orally 30 minutes prior to imaging. An unenhanced scan was obtained at 120 kV and 250 mA. The scanning layer thickness was 5 mm with a 1-mm pitch. Intravenous nonionic contrast material (1.5 ml of iopromide per kilogram of body weight, Ultravist 370; Schering, Berlin, Germany) was administered into the antecubital vein at 3 ml/s via a high-pressure syringe. Dual-phasic helical scans were obtained at 30–35 seconds (the arterial phase) and 50–60 seconds (the portal-venous phase).

### Delineation of the LNc

As a reference image, we used a set of enhanced CT images from one standard patient with a normal abdomen. The patient was selected using the following basic principles. First, CT examination of the patient was performed using a 64-slice spiral CT with a scanning layer thickness of 1-mm pitch to ensure that the relevant vessels were observed. Second, the patient had a normal abdomen and the stomach was in a half-filled state to more closely mimic the state of GC patients before neoadjuvant radiotherapy. Third, the patient had a clear anatomic abdominal structure, including the relevant vessels, which could be delineated on axial views of CT images. Based on these criteria, a 41-year-old man 174 cm in height and 64 kg in weight (BMI: 21.1) was selected as the standard patient.

LNc was defined as the central position of the metastatic LN shown in the CT images of 255 patients. Nodal grouping was also based on the 3^rd^ classification of the JGCA [26]. The processes used for delineation are as follows.

Firstly, the relevant vessels were delineated on axial views of CT images. We contoured the left gastric artery (LGA), the right gastric artery (RGA), the phrenica inferior sinistra artery (PISA), the gastricae breves artery (GBA), the celiac artery (CA), the common hepatic artery (CHA), the splenic artery (SA), the proper hepatic artery (PHA), the left gastroepiploic artery (LGEA), the right gastroepiploic artery (RGEA), the right gastroepiploic vein (RGEV), and the gastroduodenal artery (GDA), the superior mesenteric artery (SMA), the superior mesenteric vein (SMV), the middle colic artery (MCA), and the inferior mesenteric artery (IMA) on the reference CT images.

**Figure 3:**
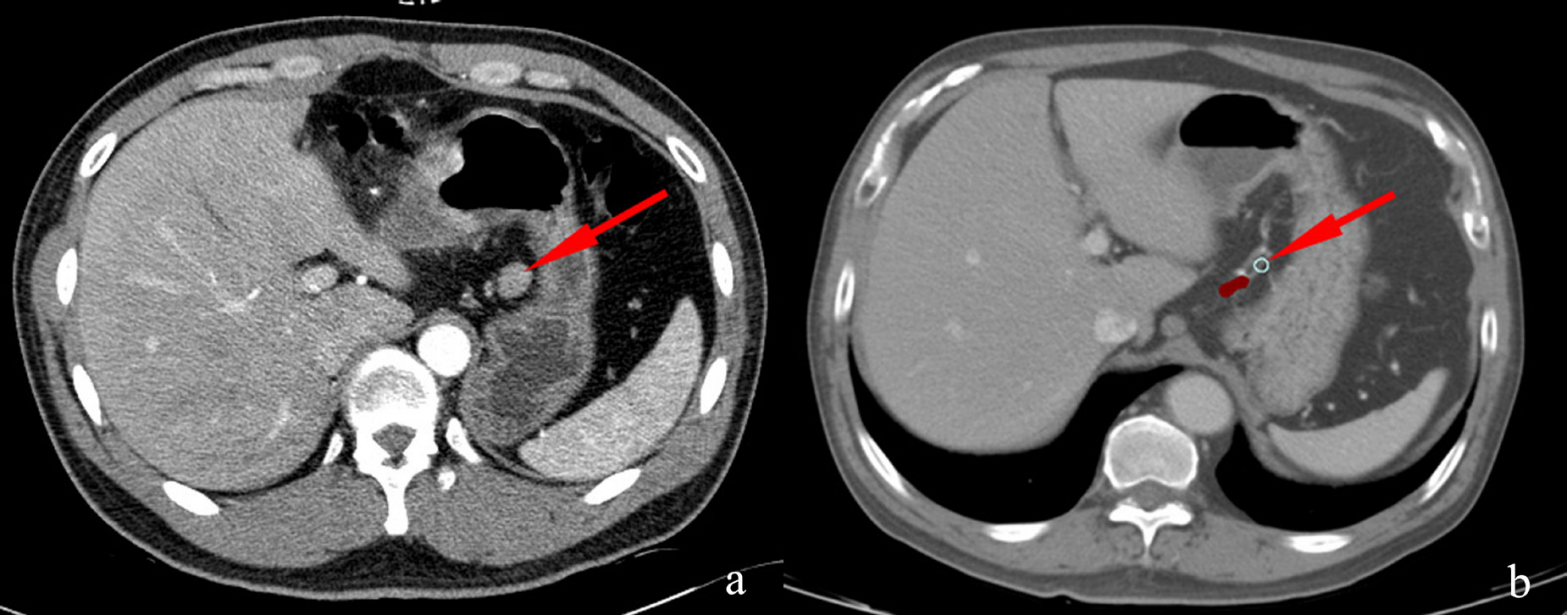
Delineation of LNc in the standard patient **(a)** An enlarged lymph node (arrow) located in the lesser curvature in a 33-year-old male gastric cancer patient. **(b)** A circle with a diameter of 5 mm (arrow) was used to replace the center of the enlarged lymph node at an equivalent location based on the relative distances to the same reference vessels and the gastric wall of the standard patient.

Secondly, we drew the 2846 LNcs on the Monaco® version 5.0 workstation. Figure 3 showed the delineation process. The basic principles were as follows. (a) We depicted the contour of the LNc at the equivalent location on the axial views of CT images compared with that in the GC patient by measuring the distance from the lymph node to landmarks such as the vascular structure, the gastric wall, the pancreas and the spleen. (b) A circle with a diameter of 5 mm was used to replace the central position of the metastatic LN to avoid the mass effect of enlarged LNs. This process is also suitable when metastasis LNs invade the vessels or the adjacent structure. (c) When the nodes mixed together, we drew the LNc of each node that was distinguishable in its respective location. Otherwise, we drew the LNc of their geometric central position. A consensus committee of three physicians, two radiologists with a gastrointestinal specialty and one radiation oncologist with three years of work experience agreed on this process.

### Image manipulation

We collaborated with the Sun Yat-sen University School of Data and Computer Science to develop image manipulation software called Medi-capture. Matrix Laboratory (2013a, MATLAB MathWorks companies in the United States) was used for image analysis and programming. A total of 2846 LNcs from the Monaco workstation were exported into 2846 files. Each file contained the space coordinate information of the structure, which can be used to determine the position of the pixels located in space and can restore the outline of each LNc in the software.

Then, the Medi-capture software, which can produce the contour and density atlas of 16 LN stations, was programmed for image analysis. First, algorithms for the contouring of the LN distribution were used to overlap LNcs located in the same cross section. The *imdilate* function, *imerode* function, *edge* function and roberts operator in MATLAB were used to process the images. Next, algorithms to calculate the distribution density of each lymph node station were used to overlap the LNcs located in the same cross section and to obtain grayscale images. A positive correlation was found between the gray value and the transparency of the color of each LN station. Twenty colors were assigned to LN stations 1–15, 16a1, 16a2, 16b1, and 16b2. The color scheme is shown in Table 2.

**Table 2:** The color scheme of the 16 lymph node stations

Lymph node station number	Lymph node station	Color swatch	Color
1	Right pericardial LNs		Rose red
2	Left pericardial LNs		Prussian blue
3	Lesser curvature LNs		Green
4	Left greater curvature LNs		Yellow
5	Suprapyloric LNs		Dark green
6	Infrapyloric LNs		Acid blue
7	left gastric artery trunk LNs		Red
8	Common hepatic artery LNs		Claybank
9	Celiac artery LNs		Crimson
10	Splenic hilar LNs		Cyan
11	Splenic artery LNs		Brown
12	Hepatoduodenal ligament LNs		Dark blue
13	LNs on the posterior surface of the pancreatic head		lemon yellow
14v	Superior mesenteric vein LNs		Orange
15	Middle colic vessels LNs		Light blue
16a1	Paraaortic LNs in the diaphragmatic aortic hiatus		Sky blue
16a2	Paraaortic LNs between the upper margin of the origin of the celiac artery and the lower border of the left renal vein		Purple
16b1	Paraaortic LNs between the lower border of the left renal vein and the upper border of the origin of the inferior mesenteric artery		Pale green
16b2	Paraaortic LNs between the upper border of the origin of the inferior mesenteric artery and the aortic bifurcation		Pink
